# Case of Impetigo Successfully Treated

**Published:** 1823-12

**Authors:** John Green

**Affiliations:** Member of the Royal College of Surgeons.


ART. X.—*Case of Impetigo successfully treated.* By JOHN GREEN, Esq. Member of the Royal College of Surgeons.

YOU were pleased to notice, in your late Journal, a case of cutaneous disease, at that time under my care: you moreover observed the great amendment which had taken place in a short time, by the method used for the gentleman's recovery. The result, "as it now rests," may perhaps interest you, and be satisfactory to your readers: with this impression, I am induced to forward to you a detail of the circumstances therewith connected, and solicit the favour of your inserting it in your valuable Journal, if you deem it sufficiently important.

I have now an opportunity of ascertaining the particulars of the case from its commencement. The gentleman who is the subject of it is about forty-five years of age: when about four or five years old he had an attack of scabies, for which he underwent the usual treatment; when convalescent, he was much bathed in the sea, with a view to strengthen him, having always been a weakly child; and to this he attributes his subsequent sufferings, as he has scarcely ever been quite free from disorder of the skin from that period, and knows that his parents were free from any such disease. When this gentleman came to me, three months ago, he was covered from the ears to the insteps with impetigo; he was unable to walk or wear his shoes; he was obliged to move with great caution; otherwise it was followed by deep cracks in the skin, which were very painful, giving issue to a watery fluid. The parts most severely affected were the legs, the perineum, the skin covering the glutea muscles, the lumbar region, between the shoulders, the axillæ, and the flexures of the arms. His complaint suffered an exacerbation generally in the spring and autumn. A short journey in warm weather always brought on fresh malignity in the complaint. At one period, he was free from any severe attack of the disease for upwards of seven years: his health was then impaired, and he had always a dry unhealthy skin; he was, however, encouraged to hope that his formidable associate was about to leave him; but it returned with its accustomed violence, and he again had recourse to medicine for relief. His perseverance in the use of remedies may be judged of by his sending back, at one time, upwards of thirteen dozen of quart bottles, which had contained Velno's syrup, and which he took at the direction of the late Dr. Willan: other different modes of treatment he followed up with similar pertinacity.

This gentleman, about four years ago, when in France, was induced to try the fumigating sulphur-bath, at a time when he was literally incased in scab. He soon began to find the good effects of these baths, and, by persevering in the daily use of them for seven weeks, the disease seemed cured. He took no medicine of any consequence during the progress of amendment. I here call it *amendment* only; as the disease, in about five months, attacked him again in its usual way, and which it has continued to do twice a year to the present period; but he invariably gets well by the use of these baths in five, six, or seven weeks. During their use, his general health always improves, taking, when needful, some aperient medicine.

When this gentleman came to me on the 1st of August, the disease showed itself in the way I have described. In the course of a fortnight great amendment had taken place, and it was about this time that the respected editor of this Journal saw the patient. The improvement had been progressive: behind the ears, the axillæ, the flexures of the arms, between the shoulders, and the lumbar region, were well; the sacrum, the perineum, the flexures of the hams, and one ankle, had still much disease in them. He declined taking any medicine to aid the progress of cure, and began to use the baths only every other day: he soon, however, returned to their daily use, as there came on a fresh access of disease, showing itself at the ankle, and in pustules on the thighs, containing pellucid matter, and an inflamed base to each; at the same time, on parts contiguous, were small watery vesicles, without much inflammation. By the daily use of the baths, this accession soon gave way; the sacrum and legs got well, but the parts pressed on whilst sitting was still very troublesome to him.

September 3d.—A slight attack of fever had confined him to the house for a few days, which soon subsided by the employment of the usual remedies. On his return to me, he complained of stiffness and pain in the groins; the glands were enlarged, and sore to the touch; he complained of a feeling of heat about the face, where, he said, from the sensation, he expected the disease to appear. On the day following the chin was covered with vesicles, attended with much heat and smarting: these vesicles, when broken, discharged ichor, gradually forming scab; there was no appearance of pustules about the face.

Fomentations, and a bread-and-water poultice at night, were applied to these parts.

Sept. 8th.—The swelling of the inguinal glands had subsided. Inflammation and soreness spread to considerable extent in the groins, but no appearance of pustule or vesicle was to be seen near to this surface: it seemed to be a distinct attack of intertrigo, accompanied with the usual discharge. Nearer to one of the knees than the groin, there were many phlygacious pustules, with yellow heads. This gentleman was so accustomed to his complaint, that his own account of its usual various changes deserved attention: he said, the appearance in the groins invariably preceded his recovery on former occasions.

Sept. 14.—A patch, the size of a crown-piece, had made its appearance a little above the right clavicle: it was of a vivid red colour, and was attended with a sensation of great heat and smarting. The legs were nearly well, but discoloured; the pustules above the knee did not threaten activity; the groins were troublesome; immediately on the sitting parts were two deep ragades, occasioning much inconvenience.

Sept. 15.—In twenty-four hours, the complaint had spread above the right clavicle across the sternum to the left clavicle, terminating in a red spot similar to the other, though not so large, and connected by a line of inflammation. The patch on the right had clusters of watery vesicles on it.

Sept. 16.—This day the whole of the parts affected on the sternum were covered with vesicles, hanging, as it were, like a necklace. From the capricious variety that this disease assumed, it became interesting, and it was seen by many of my medical friends and visitors.

Sept. 21.—The legs and thighs were well; the intertrigo less troublesome; the ragades on the seat much better; the centre of the patch on the right clavicle had become of a dead-pale colour, and many of the vesicles had disappeared; the scab on the chin in parts had fallen off, leaving a red surface, without ichorous discharge.

Sept. 28.—The complaint on the neck had become dry, with thin laminæ of scab; the seat was nearly well, but the intertrigo was still troublesome. My patient had been for some time anxious to proceed on a journey, though yet not in a fit state, and I lost sight of him for nearly a month. On his return, I found the intertrigo increased, and still some ragades on the seat. This was only what he expected, as the effects of travelling, and living less regularly than he was used to do. He again commenced the baths, which he continued for a fortnight, and got well.

I am fearful of trespassing on your valuable sheets, and have only been induced to forward this, as you expressed an interest in the result of the case: it had some peculiarities, and I think will readily be admitted as a mixed disease, combining complaints of distinct orders—*intertrigo* and *impetigo*. The affection of the face appeared to be porrigo favosa; and I thought the affection of the chest to have been conveyed by the discharge from the chin, whilst looking down on the diseased parts below, when naked. I will not take up the reader's time with an attempt to enter into the pathology of these diseases. The latter disease, writers agree, is sometimes conveyed by contagion, from the face of one person to another, and to the wrists and arms. The accurate definitions so elegantly depicted and described by the esteemed authors of our own country would lead us only to more perspicuity of expression in detailing and observing the symptoms of this class of diseases, though not materially aid us in the medical treatment; whilst the latter is the principal object of the dermologists of the continent. Their mode of cure with the fumigating baths is applicable to every variety of skin-disease, and the success attendant on their practice certainly deserves more attention in this country, where at present fumigating baths are principally in the hands of persons not of sufficient discrimination for the practice to be equally successful. It is only when members of the profession adopt more exclusively the treatment of cutaneous disease as a branch of practice, that we shall be enabled to combat them with well-grounded hope of success. No man's knowledge of these complaints ever exceeded that of the late Dr. Willan; but he was extremely cautious in recommending his modes of cure, and I believe would never particularise any medicine as being most advantageous. It was a frequent expression of his, "that a shade even of difference in the character of the disease seemed to make that medicine quite inert, which, without such shade of difference, had before proved of great efficacy." As far as my knowledge of the remedy would authorise me to speak, it appears the most valuable auxiliary that has yet been introduced to practice: by saturating the system with sulphur, (at all times a medicine of good repute,) together with the increased temperature the body of the patient is submitted to, we bring on quite a new action on the various secreting organs, by which means many latent diseases are carried off.

Independently of the praise due to the remedial powers of these baths in skin-diseases, my experience in them prompts me to acknowledge their efficacy as a tonic. In no one case that has come under my care since I have introduced these baths in Bury-street, when they have been taken even for a short time, have they ever failed in restoring strength in a way that I have never seen effected by medical treatment alone; but it must be borne in mind, that they are inadmissible in diseases of the nobler organs of the body.

To say thus much of it must be satisfactory to many of the profession, for cutaneous complaints loudly called for such an auxiliary: those complaints, as most have found, are too mixed, too changeable, and capricious, in their nature, and require too much modification of treatment, for any single remedy to be sufficient in all cases. The mode here spoken of, on many accounts, deserves the preference, which it is unnecessary now to dwell on: the general health is improved by it; it is perfectly safe when administered with discrimination; restriction from customary occupations is unnecessary; and, where the surface is generally affected, the disagreeableness of unguental applications is obviated.

I am not without hopes of being enabled to keep off this patient's malady by using the baths before the expected relapse, or on the show of any premonitory symptoms, and in the interim trusting to his frequent use of a most complete portable vapour-bath, invented by my friend Captain Jekyll, of the Navy. This bath, with other advantages, can be applied locally, and the volume of vapour or heat may be increased or diminished, and apportioned to any part of the body that may be required. It may be used with advantage in all cases where a warm-water bath is indicated; and, when taken as a general bath, the head is not included, which must be desirable in those circumstances where the surface of the body is affected with ulcer or disease. The dresses appertaining, together with the whole apparatus, is packed in a moderate-sized hat-box, as may be seen by any of the profession, at No. 5, Bury-street, St. James's.*

---

* See the Frontispiece to this Number; and our "INTELLIGENCE" for a description of the Engraving.

