# Errata

**Published:** 1824-01

**Authors:** 


ERRATA.

In our last No. p. 469, for *John* Green, Esq. read *Jonathan* Green, Esq.

